# Endoreplication—Why Are We Not Using Its Full Application Potential?

**DOI:** 10.3390/ijms241411859

**Published:** 2023-07-24

**Authors:** Izabela Kołodziejczyk, Przemysław Tomczyk, Andrzej Kaźmierczak

**Affiliations:** 1 Department of Geobotany and Plant Ecology, Faculty of Biology and Environmental Protection, University of Lodz, Banacha 12/14, 90237 Lodz, Poland; izabela.kolodziejczyk@biol.uni.lodz.pl; 2 The National Institute of Horticultural Research, Konstytucji 3 Maja 1/3, 96100 Skierniewice, Poland; przemyslaw.tomczyk@inhort.pl; 3 Department of Cytophysiology, Faculty of Biology and Environmental Protection, University of Lodz, Pomorska 141/143, 90237 Lodz, Poland

**Keywords:** endoreplication, endocycles, polyploids, polyploid plants, benefits of polyploid plants

## Abstract

Endoreplication—a process that is common in plants and also accompanies changes in the development of animal organisms—has been seen from a new perspective in recent years. In the paper, we not only shed light on this view, but we would also like to promote an understanding of the application potential of this phenomenon in plant cultivation. Endoreplication is a pathway for cell development, slightly different from the classical somatic cell cycle, which ends with mitosis. Since many rounds of DNA synthesis take place within its course, endoreplication is a kind of evolutionary compensation for the relatively small amount of genetic material that plants possess. It allows for its multiplication and active use through transcription and translation. The presence of endoreplication in plants has many positive consequences. In this case, repeatedly produced copies of genes, through the corresponding transcripts, help the plant acquire the favorable properties for which proteins are responsible directly or indirectly. These include features that are desirable in terms of cultivation and marketing: a greater saturation of fruit and flower colors, a stronger aroma, a sweeter fruit taste, an accumulation of nutrients, an increased resistance to biotic and abiotic stress, superior tolerance to adverse environmental conditions, and faster organ growth (and consequently the faster growth of the whole plant and its biomass). The two last features are related to the nuclear-cytoplasmic ratio—the greater the content of DNA in the nucleus, the higher the volume of cytoplasm, and thus the larger the cell size. Endoreplication not only allows cells to reach larger sizes but also to save the materials used to build organelles, which are then passed on to daughter cells after division, thus ending the classic cell cycle. However, the content of genetic material in the cell nucleus determines the number of corresponding organelles. The article also draws attention to the potential practical applications of the phenomenon and the factors currently limiting its use.

## 1. Introduction

### 1.1. Basic Differences between the Cell Cycle and the Endocycle

#### 1.1.1. Classic Cell Cycle

The standard cell cycle is divided into two periods: (1) the interphase, with the phases G1, S, and G2 and (2) cell division, either mitosis or meiosis. Initially, each new cell is in the G1 (Gap 1) phase [1,2]. Then, the content of genetic material in the cell nucleus amounts to 2C, i.e., it reaches the basic value in vegetative cells [3,4]. In generative cells, it is 1C [5,6]. As mentioned above, the DNA content of the cell nucleus is reflected in the size of the entire cell; therefore, in the first phase of the cell cycle, the cell volume is low.

The next step in the classical cell cycle is the S phase (synthesis), which means successive cell growth and one round of DNA replication. The amount of genetic material is gradually increased as the original DNA strand is copied, the C value goes from 2 to 4 in somatic cells, and from 1 to 2 in reproductive cells [7].

**Figure 1:**
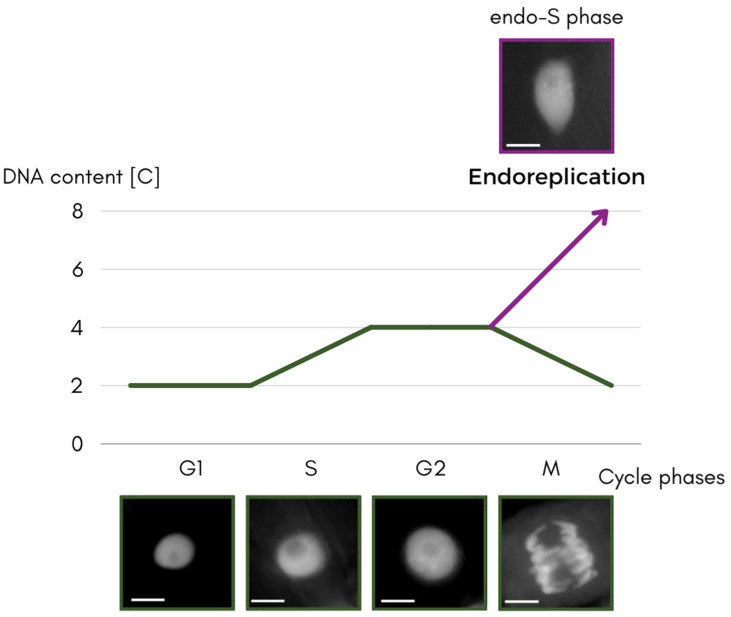
Scheme of the separating the paths of the classical cycle and the endocycle, through the cycle phases with DNA content expressed in universal units [C] of the cell nucleus changes. Nuclei of the apical part of roots of *Vicia faba* ssp. *minor* seedlings stained with 4′,6-diamidino-2-phenylindole (DAPI) and converted to grayscale. Scale bar is 10 μm.

The S phase is the gateway to understanding what happens in the non-classical type of cell cycle—the process of endoreplication described here (Figure 1). It has features in common with the cycle that ends in mitosis, but we will return to this and the differences between them later on in this review.

**Figure 2:**
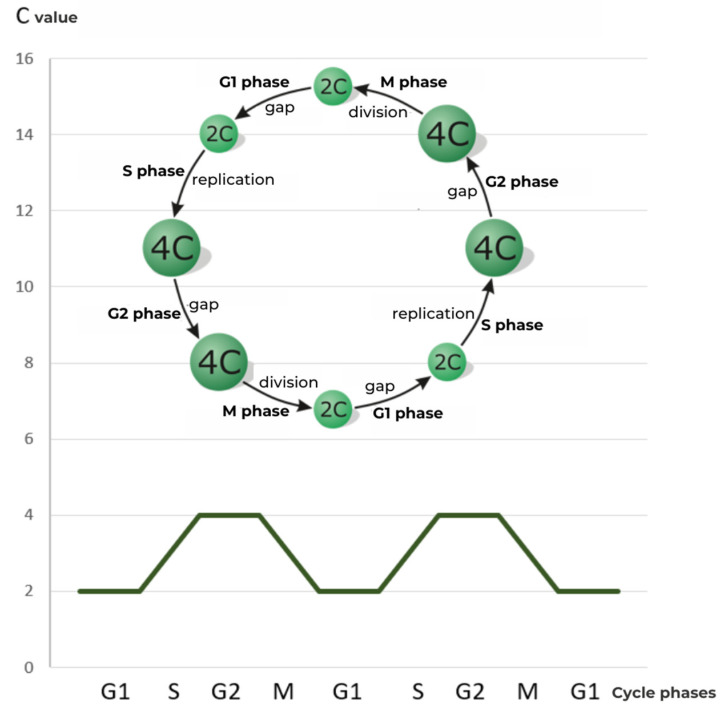
Scheme of the cell division cycle, with G1, S, G2, M phases, and relative DNA content expressed in universal units [C].

In the phase ending the classical cycle interphase, G2 (Gap 2), the cell reaches its final size, adequate to the content of the cell nucleus (Figure 2) [3]. Now, after one round of replication, the amount of DNA is 4C in vegetative cells and 2C in generative cells [6].

During the classical cycle ending with division, the value of C, characteristic of the G2 phase, decreases to the value of C, which is typical of the G1 phase. Then, the cycle begins anew [5]. There are cases where unreduced gametes (e.g., diploid instead of haploid) are formed during meiosis; when combined, they create a new organism that is immediately polyploid [6,8]. However, it is a different type of polyploidization known as species polyploidy or auto-polyploidy [9,10,11,12], which will not be discussed extensively here, unlike the organo- or tissue-specific endopolyploidy, which occurs during the lifetime of an individual, resulting from endoreplication. We will focus here on the latter pathway.

#### 1.1.2. Foundations of Endoreplication

Young plant cells are characterized by high proliferation. After some time, those that lie outside the meristems begin to differentiate, which leads to the formation of tissues [5,13].

**Figure 3:**
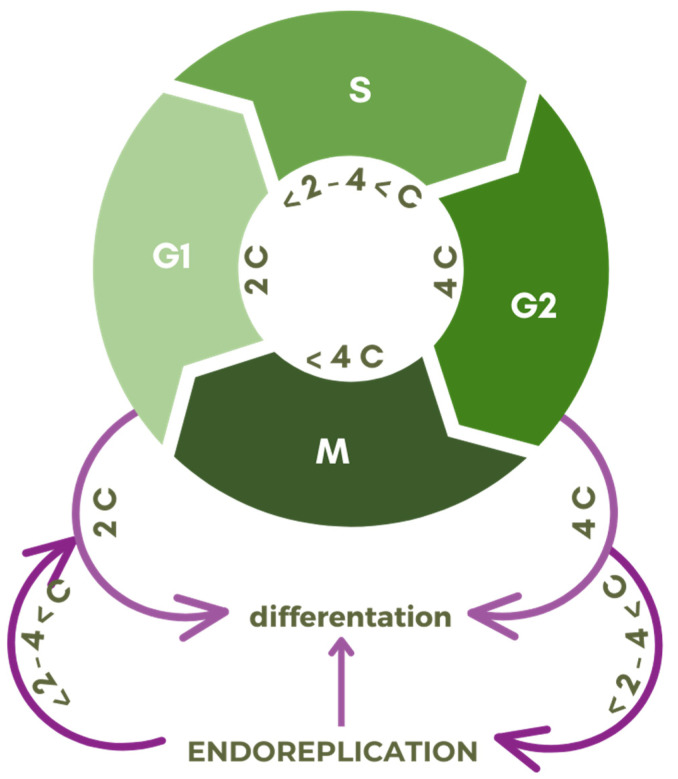
Scheme of transition points of the cell division cycle with G1, S, G2, M phases, and relative DNA content expressed in universal units (C) to the endocycle (endoreplication).

Differentiation (Figure 3) is associated with numerous changes within the cell in terms of its morphology, function, and biochemical arsenal [3,14,15,16]. The reason for such transformations is the need to shape an appropriate pool of matrices for the production of cellular components [17,18,19]. Behind the acquisition of the former is a different expression of the desired genes, and in the case of endoreplication, increasing their availability by copying the entire genome, not once, as in the case of the S phase of the classic cell cycle, but multiple times [1,20,21,22].

Endoreplication, called the endocycle, is a succession of G and S phases during which the cell grows and increases the level of its genetic material, which is expressed in the multiplication of the C value [3,4].

**Figure 4:**
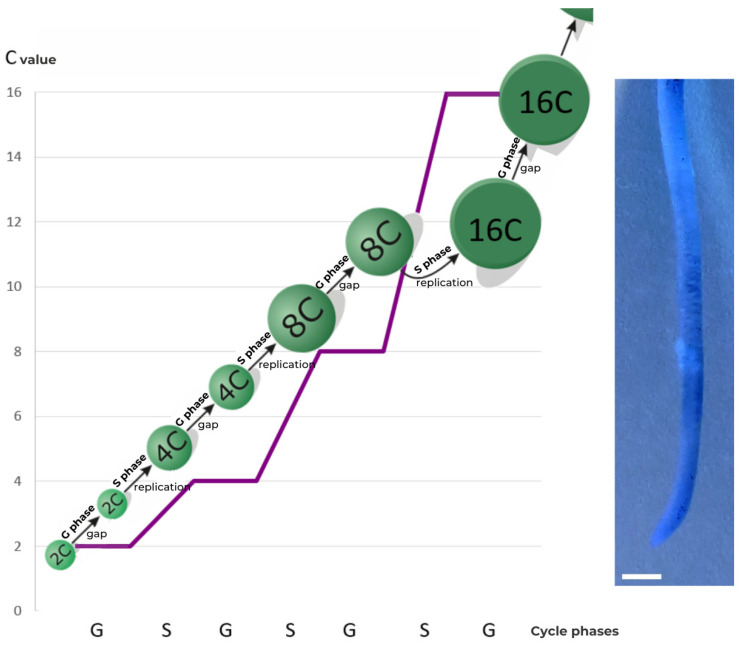
Scheme of the endocycle phases and relative DNA content expressed in universal units [C]. Image shows the apical part of root of *Vicia faba* ssp. *minor* seedlings, converted using PicosmosTools 2.6.0.1 software (first free version published in Softonic on September 29, 2015, by “Free Time”, England). Scale bar is 2 mm.

Endocycles are therefore described as division-free cycles, since the latter would normally reduce the C value by half. This does not occur in the case of endoreplication. Therefore, the cell subjected to it becomes polyploid and has a cell nucleus with an increased DNA content, often many times over, as compared to its basic content (Figure 4).

#### 1.1.3. Endocycle Types

Endocycles come in several forms, which can be distinguished by their different courses and the obtained C value. As the papers [1,23,24,25] report, these forms include endoreduplication, endomitosis, incomplete replication, and amplification.

##### Endoreduplication

The occurrence of endoreduplication in cells can be recognized by two symptoms. First, in the interphase, there is an increase in the volume of chromocenters, but there is no increase in their basal number. Second, after the interphase, the cell produces chromosomes that are ostensibly similar to those formed in the classical cycle. The chromosomes even align in the equatorial plane during endoreduplication, but they never separate. This gives rise to polythene chromosomes, i.e., structures consisting of many strands of DNA. They can be composed of multiple, even hundreds of, sister chromatids, which depends on the number of rounds of replication within endocycles. During endoreduplication, no karyokinesis or cytokinesis occurs, the spindle does not function, and no phragmoplast is formed [23].

In low polyploid cells, endoreduplication may be preceded by other types of endocycles. The occurrence of endoreduplication has been described in the seed endosperm and suspensor of many species. And it is, along with endomitosis, the most common cause of polyploidization.

##### Endomitosis

Endomitosis is a form of endocycle that closely resembles the standard cell cycle. In its course, DNA multiplication takes place, which is visible in the interphase: the number of chromocenters is doubled but their volume is not enlarged. Their number increases exponentially, corresponding to the number of replication rounds performed.

When genetic material condenses, chromosomes analogous to mitotic ones become visible. They line up within equatorial plate, as in the metaphase, and due to the action of the karyokinetic spindle, sister chromatids are separated as in the anaphase. However, they do not reach the poles of the cell as in the telophase, and the karyokinesis is only provisional and not accompanied by cytokinesis [1]. Other sources say that the karyokinetic spindle does not arise during endomitosis [26], although such a course of events would cover to some extent the similarity of endomitosis to mitosis.

In each case, one nuclear envelope is finally rebuilt, inside which all chromosomes are locked. A cell is therefore equipped with a multiplied number of chromosomes, centromeres, and nucleoli but still has one cell nucleus.

The aforementioned similarities to the division occurring in the classical cell cycle allowed scientists to distinguish four phases of endomitosis: endoprophase, endometaphase, endoanaphase, and ostensible endotelophase (ostensible due to the fact that this phase is the most divergent from its mitotic counterpart). Therefore, endomitosis could be compared to a cell cycle in which mitosis occurs but is not completed by division [1,25].

##### Incomplete Replication

During incomplete replication, genetic material is duplicated, but in an incomplete volume. Hence, the phenomenon is also referred to as underreplication. This means that the cell nucleus, after the process of incomplete replication, reaches the content of genetic material always slightly below a full multiple of C [2,24].

It depends on the needs of the plant, which goes through various stages of development during its life [2]. The functions performed by cells often require an enhanced expression of only part of the genes present in the genome, while others are redundant at this time. Incomplete replication is economical in the sense that it does not copy DNA strand fragments that will not be ultimately used. Another significant saving is the fact that during the synthesis of deoxyribonucleic acid, taking place as part of incomplete replication, the area of heterochromatin is very frequently omitted [24].

What is observed in this process are fragmentary polythene chromosomes. Incomplete replication may start upon the cell entering the endocycle, or it may occur after some degree of ploidy has been achieved.

##### Amplification

Amplification, or overreplication, is analogous to the process described above; however, it consists of the additional synthesis of only selected fragments of DNA strands to increase the chances of plant survival in unfavorable environmental conditions or the expression of selected genes that are needed, for example, at some period of development [2,27]. During amplification, the replication rounds include short fragments of genetic material or single genes [24].

In cell nuclei subjected to cytometric tests, the C-value is always slightly above polyploidy when amplified. However, it is difficult to show this unequivocally because the same values may also mean that the cell undergoes a replication process or a different type of endocycle [24,27]. Hence, to demonstrate that the genetic material has indeed been amplified, preliminary studies should be confirmed via analyses carried out at the molecular level.

## 2. Endoreplication in a Molecular Shortcut

It took scientists a very long time to elucidate the molecular basis of endoreplication, but now it seems to be quite well understood, especially due to the use of the model organism *Arabidopsis thaliana* [28,29].

An inherent element of the mechanisms governing endoreplication is the abandonment of mitotic divisions—the action of factors promoting (Table 1) the course of this type of cell cycle. To understand the phenomenon, we must start with CDK-Cyc (kinase–cyclin) complexes. Endoreplication depends on a decrease in the activity of these complexes [30].

Studies on *A. thaliana* mutants deprived of the activity of CDKB1 kinase, known to promote mitosis, which combines with A2 cyclins, have shown that endoreplication occurs in these organisms. This proves that its activity is inhibited if endoreplication is to occur [28,29].

However, how are the indicated complexes in plants deactivated without human intervention? This happens due to the presence of factors that are CDK inhibitors [30], e.g., SMRs, which are up-regulated upon the entry of the cell into the endocycle. The second possibility is the stimulation of degradation of cyclins [31], on which mitotic cycles depend. The anaphase-promoting complex/cyclosome (APC/C) plays a role here (Table 1) [32].

No matter which version we consider, the regulation of these modulations of the cycle is mediated by protein complexes, transcription factors, or at the level of transcription itself, subject to changes depending on, e.g., phytohormones or biostimulators [33].

MED 16 (Table 1) is one of the transcription factors responsible for switching between cycles. A subunit of the MED16 complex has been described as affecting the circadian rhythm, flowering time, the occurrence of cooling tolerance, and the maintenance of Fe homeostasis. The latter features are in particular attributed to polyploid plants [33]. This is related to the ability of MED16 to reduce the expression of CCS52A1 from CELL CYCLE SWITCH52 (CCS52A1/A2) [34]. CCS52A1/A2 is so important that it activates the aforementioned APC/C. *A. thaliana* MED16 mutants show a higher level of ploidy than the wild type, which proves that MED16 is required for normal cell growth and the presence of endoreplication, since it inhibits APC/C activity at the point required in mitotic cycles.

LATE MERISTEM IDENTITY 1 (LMI1) [32] is also the cause of endoreplication, which was proven based on studies on *A. thaliana*, in which the proportion of the leaf was variable, and beneficial after endoreplication, depending on the activity of this factor. LMI1 is important because its activating power has been demonstrated in relation to WEE1 (Table 1), which is responsible for the inhibition of mitotic divisions. This protein is crucial for the occurrence of endoreplication, although its importance has been questioned [35].

Other reports indicate a significant role of SOG1 (SUPPRESSOR OF GAMMA RESPONSE 1) in promoting endocycles (Table 1), which was also tested on *A. thaliana* mutants subjected to salinity stress. The occurrence of endoreduplication was important here because, apart from programmed cell death, it ensured the survival of plants in unfavorable conditions, allowing for a greater volume of cells in the above-ground part and for an increased branching of trichomes. Due to stress-induced DNA damage, cell cycle G2/M checkpoints [36] (otherwise S/G2 [37]), played a key role here, enabling the maintenance of genome stability and preventing mitosis by reducing the expression of CYCB1;1, CDKB1;1, and CDKB2;1, as well as by increasing the expression of WEE1, CCS52A, and E2Fa, allowing for the induction of endoreduplication, involving just SOG1. This was possible thanks to a rather complex signaling network, using ATM (ATAXIA-TELANGIECTASIA-MUTATED) and ATR (ATAXIA-RAD3-RELATED), i.e., specific kinase “sensors” that alert the organism to damage. On the other hand, the SOG1 transcription factor acts as an intermediary in the transduction of signals from ATM and ATR, launching a further cascade of reactions leading to unwinding in the form of repair of DNA strand breaks, endoreduplication, and programmed cell death. It seems that these processes interact and overlap to some extent to give the plant the best survival effect. This process occurred in line with SOG1 overexpression, while sog1 individuals with suppressed genes were almost completely deficient in it and did not adapt to the conditions of saline stress and the accompanying oxidative stress [36,38].

The aforementioned E2Fa is a transcription factor causing additional DNA replication in cells, which is necessarily correlated with inducing the transcription of genes responsible for the S-phase and activating the cycle-specific CDK complex for this period, as described, for example, in studies on changes in maize endosperm after the start of endoreduplication [39]. On the other hand, *A. thaliana* mutants with E2Fa-DPa silencing underwent some inhibition at an early stage of development because of a reduction in the rate of cell division and the absence of endocycles. This provided an insight into the importance of a controlled exit from the classical cell cycle, which determines the further development of the plant and is associated with differentiation. In turn, plants over expressing E2Fa were characterized by silencing M-phase-specific CDK activity and a drastic increase in S-phase gene expression, normally inhibited by the inactivating effect of Rb on E2Fa-DP due to complex binding and an increased presence of endocycles [40,41,42].

TOP6B also plays an important role in promoting endoreplication (Table 1). Plants devoid of its activity were characterized by reduced levels of ploidy, drought stress tolerance, activity of antioxidant enzymes, relative water content (RWC), and proline but an increased concentration of malondialdehyde (MDA). In turn, *A. thaliana* specimens overexpressing TOP6B were characterized by increased growth and development of aboveground and underground organs and reached high levels of ploidy, which resulted in increased secondary metabolism, increased tolerance to stress, and all the abovementioned indicators were maintained for a very favorable level. However, transcriptome analysis revealed that genes involved in the cell cycle, transcription, and signal transduction were most often up-regulated in these mutants. However, there is no agreement as to how TOP6B would directly affect the transcription of genes encoding cyclins [41,42,43].

To sum up, the role of regulators of activity/inactivation of CDK-Cyc complexes, such as SMR, WEE1 (inhibition of CDK and APC/C), and TOP6B [30], is of key importance for endoreplication. Transcription factors, such as MED16, LMI1, SOG1, and E2Fa, regulate the expression of genes related to the course of cell cycles and determine the entry of the cell into the endocycle [35,36,38,42,43].

## 3. What Do Plants Need Endocycles for?

As already mentioned, plants have a relatively small genome. The natural environment requires plants to display a substantial ability to survive in changing and often very unfavorable conditions because they cannot avoid them after settling in a habitat [21,43,44,45,46,47,48,49,50,51,52,53,54]. This can be facilitated by increasing the number of copies of genes beyond the pool they have to begin with. These requirements can be met by endoreplication, which also occurs in response to stress and is often integrated with generalized defense systems [44,45,46,47,48,49].

This is visible in the phenomenon of allelopathy. Here, endopolyploidy enables a more efficient production of detoxifying compounds, antioxidant enzymes that protect plants against the effects of substances released by other organisms, but also facilitates the formation of allelopathins [55,56,57,58,59,60,61,62]. Plants are therefore more adaptive by being able to alleviate stress and not suffer harm and/or be more “aggressive” by producing allelopathic compounds [44,45,46,47,48,49,62].

Thus, the goal of endocycles, which is to obtain copies of genes necessary for passive and active defense against biotic and abiotic stress, is justified [21,22,43,46,58,63,64,65,66,67,68,69,70,71,72,73]. Scientists see endoreplication as a specific way of controlling gene expression, which is revealed particularly in situations in which the plant needs many copies of definite genes for more efficient transcription and translation, e.g., to more vigorously counteract infections or stimulate beneficial interactions [72,74,75]. Not only do plants have a better chance of survival in the environment, but they also increase their competitiveness in terms of population and individual competition.

The competitiveness of endopolyploid plants is determined by the rapid enlargement of cell size, which is sometimes associated with the action of some phytohormones [62], and the accelerated differentiation of cells, which allow plants to reach particular stages of development at a faster rate [76,77]. This is not without significance, because the earlier the stage of development, the more susceptible the plants are to the negative influence of environmental factors. Their endurance appears to be in part dependent on the time of activation and the degree of endocycle occurrence during plant development.

Endoreplication is involved both in plant development and in maintaining the proper course of physiological processes [77]. Some sources even claim that endoploidy is essential in supporting development. The delivery of nutrients and building elements, for example, to a developing embryo [19,65], as described in the example of the polyploid endosperm of maize, is based on endoreplication. Cells after endoreplication are able to meet the high metabolic requirements of the embryo in terms of obtaining building materials and energy resources during embryogenesis [13,65]. The tasks of the polyploid endosperm of maize include, among others, the rapid initiation of starch production or protein synthesis based on multiplied mRNA, and the development of some place for storing the nucleotides needed by the germinating seed to provide sufficient initial resources for the growing seedling. The importance of endoreplication in this case was shown by an experiment in which the process was interrupted, which led to embryo lethality [64,66].

Endoreplication, being a path of cell differentiation (Figure 3), leads to obtaining specific and desirable functions and features in the tissues. Through it, cells reach maturity but also the aforementioned ability to activate a more efficient nutritional machinery and defense mechanisms. In turn, disorders in endoreplication can cause the organ to malfunction and make it more susceptible to pathogenesis. Hence, the claim that endocycles are closely associated with development and even necessary for the proper growth of plants in which they occur [10,65,66].

Upon gaining a greater content of protein components, i.e., basic functional and causative units in the cell, polyploid plants have a more efficient metabolism [1]. This leads to an overproduction of substances responsible for their taste, flavor, and visual qualities. For example, fruits with polyploid cells are of higher quality and are sweeter and juicier [5]. Flowers with polyploid cells are characterized by a more intense color and smell. This is due to improved metabolic management, which increases the production of, for example, aromatic oils and dyes. As a result, flowers become more attractive to pollinators and acquire superior ornamental qualities, while fruits become more attractive for animal consumption promoting seed dispersal, as well as for cultivation by humans [11,67].

In addition, the faster growth of organs such as roots (enlargement of the absorbent surface) [68] and leaves (enlargement of the photosynthetic surface) enables a more efficient absorption and production of the required ingredients. The enlargement of organs is often associated with the creation of additional space for the storage of substances and, therefore, the possibility of maximizing their subsequent processing. These are changes that allow the plant to increase in size. It should be remembered, however, that the size of organs and tissue volume are not unregulated, but rather are controlled by balancing between the number of polyploid cells and the rate of proliferation [9,69].

## 4. Evolutionary Role of Endoreplication

Discussing the connection between evolution and endoreplication, we should start with the notion of endopolyploidy, which is an integral part of the developmental program of eukaryotic cells. One of its types, with the complete omission of mitosis, is endoreplication [1].

The inclusion of endoreplication in evolutionary transformations is also due to the deep anchoring of its molecular base among those responsible for the control of the classic cell cycle and easy switching between the two cycle models [70]. The general patterns and factors that affect changes in genome size taking place in the course of evolution are known, but their mechanisms in plant organisms have not been fully established to date. An answer to the questions posed here may be the polyploidization resulting from endoreplication [71].

Typically, cells in plants species with large genomes do not enter the endoreplication pathway or exhibit lower levels of ploidy than those in species with small genomes. Here, a significant example is *A. thaliana* [72]. However, in this species, endoreplication also occurs mostly in several types of cells, e.g., in trichomes, ectoderm, and endosperm [73].

Evolutionarily, the size of the genome matters, as does the variability of its size. These factors are correlated with the structural components of cells and their volume [16,74] and the morphological shape of cells, while the rate of cell division is important in the adaptation of the organism to survival challenges [75,76].

Most plants show moderate levels of ploidy [17], but this common phenomenon is related to tissue-specific polyploidy and organo-specificity. Evolutionary and ecophysiological adaptation can be discussed when considering the durability of organs [77], their growth time, as well as survivability and fulfilling vegetative [70], generative functions, and especially the production of nutrient-rich seeds needed for the next generation [78]. Beneficial and quick effects in this respect are obviously brought about by endoreplication, which can thus be linked to adaptive evolution [79]. Species that are uniquely adapted and more resilient have an increased genome size that provides them with a more efficient metabolic machinery [62,63,64,65,66,67,68,69,72,73,80], responsible for the ability to maintain a high level of nutrient management [3,6,7], which is great for the relationships taking place in orchids organisms [64,73,81].

This is well-documented, e.g., in orchids, which provide an interesting example of the evolutionary importance of endoreplication. Orchids are plants with huge phenotypic diversity and plasticity depending on habitat conditions. They occur in the tropics and at high altitudes, which means that they can withstand hostile environments, but at the same time, they are cosmopolitan species [72,82]. Their evolutionary success was previously linked with an epiphytic form of growth, co-evolution with pollinators, and specific metabolism. However, recent research on orchids has shown variability in their genome size associated with partial endoreplication, which has certainly affected their adaptive potential [77,82,83,84].

In such extreme cases, the resulting cell polyploidy is responsible for the exceptional invasiveness of species and their spread, which is obviously related to resistance to or tolerance of environmental stressors [79,85,86,87]. This has also been demonstrated in plants found in areas prone to seasonal droughts [88], with exceptionally cold winters or high annual temperature fluctuations [55]. Therefore, the role of endoreplication in creating a greater possibility for the plant to survive unfavorable environmental conditions, and even severe stress, is evolutionarily important.

## 5. The Universality of Endocycles

Endoreplication is a common phenomenon in the plant kingdom. The content of DNA in polyploid cells usually ranges between 4 and 32 C, as in *A. thaliana* [60], often reaching 64 C, but there are also some exceptional record holders, such as *Arum maculatum*, in which endosperm cells were found to have 24,756 C.

Endocycles have been detected in high metabolic activity cells, e.g., in bean embryo suspensor cells (4096 C) [89], as well as those subjected to differentiation, in developing xylem or highly specialized cells, such as vanilla rapid crystal idioblasts, which also reach considerable sizes [90]. The value of 512 C is common in suspensor and endosperm cells of seed, and 256 C has been found for the nuclei of basal cells of hair anthers in *Bryonia* sp. [77,91], trichome-forming cells in *Elodea canadensis* and *A. thaliana* [88,92], cotyledon cells in many species, as well as for whole tissues, e.g., in the leaf epidermis of *A. thaliana* [5,93,94] and *Phaseolus vulgaris* [89,95], in the parenchyma of orchid seedlings [80,96], and the endosperm of *Zea mays* grains [70].

Most often, it is expansion that is behind these adaptations/functions of cells following the endoreplication pathway. Cells that differentiate into trichomes are part of the development plan [92]. In *A. thaliana*, the cells (except meristems) of developing seedlings also undergo rounds of re-replication, resulting in a genetic material content of 32C [97], while attaining appropriate sizes and shapes [3,14]. Of course, endopolyploid cells may be located between adjacent diploid cells. This occurs in the *A. thaliana* leaf epidermis (2C–64C) [93].

Many more examples could be given as the phenomenon of endopolyploidy is widespread in plants. Numerous studies focused on endocycles have led to the conclusion that most often it is the older tissues of a plant that possess a higher C value. Research on cucumbers has shown that endoreplication takes place during the successive stages of plant development [2,91]. This also applies to plants with relatively extensive genomes, such as the aforementioned maize, as well as tomatoes and potatoes. Scientific studies reporting on systemic somatic polyploidization suggest its organo-specificity [79].

## 6. Endocycle Limitations

Endopolyploidy allows plants to adapt to adverse environmental conditions, but it is not the only and final “choice” that is always good.

We have already established that endocycles are correlated with various biotic and abiotic factors, and if they counter them, they ensure the survival of the plant [92,93,98]. However, what happens if the conditions to which the plant has adapted change? Polyploidy is not easy to reverse. The limitations of proliferation (the balance between proliferation and endoreplication), multipotency, and plasticity can be disastrous [31,98].

The same goes for every single cell. Endocycles beneficial to one type of cell will not necessarily benefit another type of cell. For example, endopolyploidy could disturb the functions of cells constituting stomata, and so they remain diploid [99].

In addition, despite the aforementioned reduction in the cost of producing cellular structures, endoploidy may become too demanding due to the increased metabolism of cells that possess it. This is mainly related to the uptake of nitrogen and phosphorus, evidenced by, for example, the low presence of polyploid plants on poor soils (these elements are not abundant in standard soils) [91,92,98]. On the other hand, the increased hydration of endopolyploid cells (the additional cost of their formation) favoring size enlargement is beneficial as long as the plant accumulates water, as is the case with succulents [99]. Endoreplication is therefore beneficial where its costs are compensated.

Another tradeoff is that endopolyploid cells have a lower surface-to-volume ratio, which is reflected in a reduced membrane surface available for the reactions that require it and the efficiency of intracellular transport [99].

However, contrary to appearances, the greatest limitation of endoreplication is its very foundation, i.e., genetics and the cell cycle regulators related to it. They occur in large numbers and are characterized by a diversity of alleles and interactions depending on the taxon [4,71,95,100]. The proper harmonization of all this machinery is quite challenging, which limits the incidence of endocycles [101,102].

## 7. Difficulties in Exploiting Endoreplication by Humans

Endoreplication has a great potential and enables plants to cope with stress and grow properly. The question is: why do we not use this phenomenon for our purposes, in a way similar to genetic engineering or polyploidization?

There are still plenty of issues to explore to better understand the functioning of each branch of endoreplication. Endoreplication often correlates with, but does not appear to be a direct promoter of, growth [97]. Therefore, endoreplication itself will not always be directly reflected in the expected final effect, e.g., an increase in organ size.

Methods of artificially inducing endoreplication and controlling its course still cause some problems. The tissue area affected by endoreplication may be very differentiated, with different ploidy of nuclei. Also, endoreplication characterizing one species may be very diverse and specific. Endoreduplication progression in the endosperm differed significantly among the 10 rice cultivars researched by Kobayashi in 2019 [101].

To sum up, there is still much to elucidate in the area of endoreplication. Now, we know too little to obtain direct production profits outweighing the inputs.

## 8. Discussion and Conclusions

Endoreplication is a modified cell cycle which commonly appears in plants [1,2] and animals organisms [103]. It consists of genome replication without cell division [1,2]. This process is controlled by several factors that are also important for the cell cycle [5]. A key mechanism of the process, which occurs before the S phase of the cycle, is the formation of a pre-replication complex (pre-RC) [104].

It is complexed with replication origins by the association of the origin recognition complex by Cdt1, Cdc6, and finally MCMs, allowing DNA replication to start. It is a conserved complex in eukaryotes, which is able to receive signals and help organisms to adapt to the environment in many ways [86,103].

It should be noted that all signals might be factors influencing genetic and metabolic changes. Some abiotic factors, such as irradiation, exposure to H_2_O_2_, suboptimal temperatures, light and water conditions, salinity, and heavy metals, induce replication stress, resulting in strand/strands breaks. The most important effect of replication stress is related to changes in the metabolic level. Cells have several mechanisms for countering adverse effects. One of them is endopolyploidy, which occurs through DNA multiplication, positively affecting transcription efficiency and resulting in an increased expression of metabolic and stress-reducing genes [41].

Endopolyploidy helps obtain specific desirable functions, as well as features, in tissues. It takes part in allelopathy, plant development, maintaining the proper course of physiological processes, and the rapid enlargement of cell size (and thus, faster organ growth). Examples of such a strategy include the formation of root nodules in *Papilionaceae* plants. Furthermore, the root cortex cells of those plants contain nuclei with multiplied genetic material enabling control of the growth of the main and lateral roots without the need to increase the number of cells.

As a result, organisms have fully multiplied nuclei (endoreplication), incompletely replicated or amplified nuclei, as well as cells after endomitosis.

A crucial role in controlling all processes of growth and development, including endoreplication, is played by plant hormones. This was confirmed in *A. thaliana* roots, as it was found that ethylene (ETH), playing a pleiotropic function, is responsible for modulating the mitotic activity of cells in QC (quiescent center). Thus, it seems that ETH may be important for the overall control of the replication process, depending on whether cell division or endoploidy occurs. Extensive studies have shown that the transition from proliferation to endoreplication involves gibberellins, auxins (AUXs) and cytokinins (CKs). AUXs and CKs appear to be critical in controlling the process. While AUXs are responsible for blocking the induction of endoreplication by CycA2;3, CKs stimulate this process through ccs52A1 [105].

Nevertheless, further studies seem to be necessary to link the aforementioned role of ethylene with the hormones. The recognition of these relationships could be used to increase the level of endoreplication in plant organisms without the need to involve genetic transformation. This would reduce the problem of social acceptance of genetically modified organisms and increase alternative methods of biostimulation.

Progress in research on endoreplication may take place as part of activities related to broadly defined genomics. The clue to such reasoning was the extensive mapping of nuclei at various levels of ploidy and the discovery of the spatio-temporal control development of plant tissue as was shown during analyses of *A. thaliana* roots. In addition, the genes involved through their transcripts in individual reactions to a given stress or triggering factor may be determined. Examples of such reactions include physiological responses (rapid seed germination), biochemical activity (overproduction of anthocyanins), or structural changes (cell wall thickness). In this respect, sequencing methods are important, as interpreting the mechanisms of transcriptome can provide precise answers to the following question: How can plants cope with different environmental conditions? Australian orchids provide a prime example here: while changing climatic conditions have limited their habitat to the coastline, their species continuity was maintained [54,106].

It is possible that in-depth research will provide a broader view of the biological significance of endoreplication in the abovementioned respects as well as of their application potential, e.g., in crops such as rice and maize [18]. Knowing what factors and in what way specifically control the occurrence of endocycles in cells, it would be possible to stimulate them in tissues desirable by humans and to strengthen them (e.g., in tomato pulp). And at the same time, crops could be made more resistant to biotic and abiotic factors in ways not involving chemical protection agents, such as herbicides.

Despite the numerous advantages for plant organisms (and indirectly also for humans), endoreplication may not easily lend itself to research. For example, a recent study by Piet et al. 2022 [102], indicates that partial endoreplication is challenging to accurate assembly of whole genome and may cause problem during sequencing.

On the other hand, it may be tempting to induce endocycles as part of the safe biostimulation of plants to impart to plants qualities desired by humans, especially since we know some easy-to-use substances that stimulate this phenomenon. This potential application of endoreplication requires further research to elucidate how we can effectively and precisely induce and maintain this process.

## Figures and Tables

**Table 1:** Factors controlling activities of CDK/Cyc, a mitotic complex, allow the redirection of the mitotic to the endoreplication cycle. Primary inhibitors and secondary inhibitors and activators of the complex.

Primary Inhibitors of CDK/Cyc	Secondary	
	Inhibitors	Activators
TOP6B		
APC/C^CS52^	MED16	
WEE1		LIM1
SMRs		SOG1

Abbreviations of the factors are explained in the main text.

## Data Availability

Not applicable.
